# 3D Magnonic Conduits by Direct Write Nanofabrication

**DOI:** 10.3390/nano13131926

**Published:** 2023-06-24

**Authors:** Sebastian Lamb-Camarena, Fabrizio Porrati, Alexander Kuprava, Qi Wang, Michal Urbánek, Sven Barth, Denys Makarov, Michael Huth, Oleksandr V. Dobrovolskiy

**Affiliations:** 1 Faculty of Physics, Nanomagnetism and Magnonics, University of Vienna, Boltzmanngasse 5, A-1090 Vienna, Austria; 2 Vienna Doctoral School in Physics, University of Vienna, Boltzmanngasse 5, A-1090 Vienna, Austria; 3 Physikalisches Institut, Goethe-Universität, Max-von-Laue-Str. 1, 60438 Frankfurt am Main, Germany; porrati@physik.uni-frankfurt.de (F.P.); kuprava@physik.uni-frankfurt.de (A.K.); barth@physik.uni-frankfurt.de (S.B.); michael.huth@physik.uni-frankfurt.de (M.H.); 4 School of Physics, Huazhong University of Science and Technology, Wuhan 430074, China; williamqiwang@hust.edu.cn; 5 CEITEC BUT, Brno University of Technology, 61200 Brno, Czech Republic; michal.urbanek@ceitec.vutbr.cz; 6 Helmholtz-Zentrum Dresden-Rossendorf e.V., Institute of Ion Beam Physics and Materials Research, 01328 Dresden, Germany; d.makarov@hzdr.de

**Keywords:** nanomagnetism, 3D nanostructures, spin waves, ferromagnetic resonance, Brillouin light spectroscopy, focused electron beam induced deposition

## Abstract

Magnonics is a rapidly developing domain of nanomagnetism, with application potential in information processing systems. Realisation of this potential and miniaturisation of magnonic circuits requires their extension into the third dimension. However, so far, magnonic conduits are largely limited to thin films and 2D structures. Here, we introduce 3D magnonic nanoconduits fabricated by the direct write technique of focused-electron-beam induced deposition (FEBID). We use Brillouin light scattering (BLS) spectroscopy to demonstrate significant qualitative differences in spatially resolved spin-wave resonances of 2D and 3D nanostructures, which originates from the geometrically induced non-uniformity of the internal magnetic field. This work demonstrates the capability of FEBID as an additive manufacturing technique to produce magnetic 3D nanoarchitectures and presents the first report of BLS spectroscopy characterisation of FEBID conduits.

## 1. Introduction

Magnonics is a rapidly developing domain of modern magnetism [1]. Magnonics deals with spin waves and their quanta-magnons as perspective data carriers for information processing [2]. Spin waves are free from Joule heating [3] and are compatible with high clock speeds and frequencies up to the THz regime [4]. Various prototype magnonic devices have demonstrated computing capability for analogue and digital data processing, including a magnonic transistor [5], directional coupler [3], and other functional elements [6,7,8]. Quantum magnonics [2,9,10,11], neuromorphic computing [12], magnon Bose–Einstein condensates [13,14], inverse design systems [15,16], and interaction of magnons with other quasiparticle ensembles [17,18,19] are among several [20,21,22,23] emerging research directions motivating fundamental investigations of magnetisation dynamics and novel materials as magnonics platforms.

Miniaturisation of magnonic structures and circuits is key for both going beyond complementary metal oxide semiconductor (CMOS) processes, the current industry standard computation technology, and realisation of single magnon-mode operation. These are the primary challenges yet to be met [2,24,25]. One solution for enhancement of magnonic circuits while maintaining the same footprint is their extension into the third dimension [21]. Three-dimensional magnonics is now attracting increasing attention [26]. This interest is further motivated by the fundamental physical phenomena associated with hierarchical systems [27], systems with magnetic frustration [28], and geometrically induced non-trivial spin textures [29]. In this regard, of particular interest are 3D curvilinear systems, which have already demonstrated applications-relevant behaviour, such as non-reciprocity and stabilisation of topological spin textures [22,30]. In a broader context, 3D nanomagnetism represents a vibrant domain of research nowadays [31,32], with exciting topology- and geometry-induced phenomena unseen in planar systems. To this end, in this work we have investigated a magnonic waveguide with 3D thickness variation to analyse the supported magnetisation dynamics, with a perspective towards future data processing applications in all-magnonic circuits. Magnonic conduits are essential components for the interconnection of logical elements in magnonic circuits, and may also contribute their own logical functionalities with shape or composition engineering [2,3].

As for experimental realisation, established methods of fabricating 3D magnonic structures include strain engineering of thin films [33] and functionalisation of non-magnetic templates [22,34]; however, these techniques are limited to producing thin film or core-shell structures. At the same time, several technologies which overcome these limitations have reached a high level of maturity in recent years, such as optical writing by two-photon lithography [35] and 3D nanoprinting by focused electron and ion beams [36,37]. Among these, focused-electron-beam-induced deposition (FEBID) is highly flexible with respect to position on the substrate, substrate material, and writing material [38], and has benefits in aspects of almost no beam-induced damage of the processed area [39]. FEBID’s flexibility and down to 40 nm lateral feature size (for magnetic materials such as Co and Co-Fe [40]) have lead to its use in research and industry, for writing electrical contacts and as the tool of choice for nanoscale mask repair [38,41].

In the context of magnetic materials, post processing techniques (post growth electron irradiation, thermal annealing, etc.) can be used to engineer the magnetic properties (saturation magnetisation, exchange stiffness, and anisotropy) of FEBID deposits [42,43]. Shape anisotropy is deliberately tuned by the geometric variation of the structure’s footprint, and in the third dimension, i.e., thickness modulation. This can be used for the development of spin–wave optic devices [44,45,46] and the manipulation of spin–wave beams. In this regard, magnetic FEBID deposits have already demonstrated functional properties: structural engineering by FIB milled small holes in Co FEBID nanodots stabilised metastable intermediate states during magnetisation reversal; applicable to development of multilevel memories [47]. Fundamental elements of magnonic circuits have also been demonstrated, including a continuously tunable spin–wave phase shifter [6] representative of a NOT gate at π phase shift [48], and nanovolcano structures as multi-mode resonators with ca. 30% smaller footprint than equivalent disk or ring type resonators which may find use in signal filtering [21]. However, while tuning of magnetisation dynamics in FEBID deposits has already been studied by microwave spectroscopy, 3D magnonic conduits with strongly non-uniform internal field have so far never been probed by optical spectroscopy based on Brillouin light scattering (BLS), as will be introduced in this manuscript.

**Figure 1:**
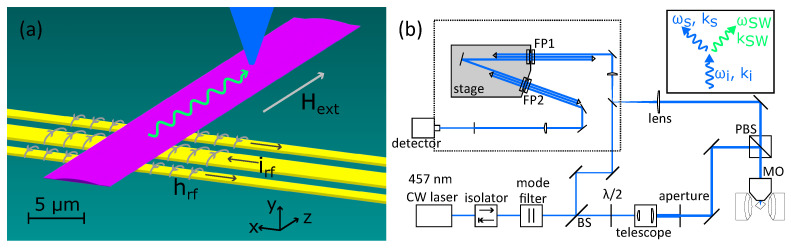
(**a**) is a cartoon representation of the employed excitation and detection scheme. Radio frequency (RF) current (black arrows) injected to the CPW (yellow) generates an Oersted field, $h_{\mathrm{rf}}$, around the conductors as indicated by the grey arrows. The Oersted field drives spin–wave dynamics in the magnonic conduit (purple) resulting in spin-wave (green) propagation along the length of the conduit. The BLS laser is indicated by the blue cone. Dimensions of the sample, CPW, and laser focus diameter are to scale. (**b**) is a schematic representation of the BLS optical path. CW = continuous wave, BS = beam splitter, PBS = polarised beam splitter, MO = microscope objective, FP = Fabry–Pérot pair. Inset is the Feynman diagram for the magnon creation interaction, demonstrating the inelastic scattering of the BLS process. Frequency and wave vector are represented by ω and **k**, respectively, for the incident photon (i), signal photon (s), and spin wave (SW).

In brief, optical probing of magnonic structures permits a spatially localised readout, which is otherwise impossible using all-electric approaches such as propagating spin-wave spectroscopy (PSWS). While there is a report of an all-electric PSWS approach which leverages varying spatial separation of excitation and detection antennas [49], optical probing allows arbitrary choice of measurement position in two dimensions across the same sample, and changing the measurement position only requires repositioning of the sample under the laser focus, as illustrated in Figure 1. Spin–wave excitation is provided by a single coplanar waveguide (CPW) in our investigation, leveraging the efficient coupling of the Oersted magnetic field around the CPW to the magnetic subsystem in the magnonic conduit. The excitation and detection scheme is illustrated in Figure 1.

Here, we compare spin–wave dynamics for 2D and 3D Co-Fe FEBID nanostructures. One structure is a flat plank with rectangular cross section, while the 3D structure is a plank pedestal supporting an axial bump along its top surface. We use BLS spectroscopy to demonstrate significant qualitative differences in the spatially resolved spin–wave resonances of the two structures as a result of the 3D geometrical engineering. Our central observation is that in contrast to the single-mode Kittel-like frequency vs. field dependence, the f(H) response for the 3D conduit is more rich and supports many spin-wave modes because of the internal field non-uniformity.

## 2. Materials and Methods

### 2.1. Fabrication

Samples are magnonic conduits deposited in a single FEBID writing phase on top of a microwave antenna. The CPW antennas were prepared on a GaAs substrate by electron beam lithography with GaAs/Ti/Cu/Au layers deposited using electron beam evaporation. Samples are written to bridge two CPW antennas. In the course of this investigation, only one CPW was contacted per conduit sample for the microwave excitation.

The magnonic conduits were grown by FEBID in a dual beam focused ion beam/scanning electron microscope (FEI Nova NanoLab 600) [50] using the heteronuclear precursor HFeCo_3_(CO)_12_. The precursor was prepared according to the method reported previously [50]. The beam voltage, current, and dwell time were 5 keV, 1.6 nA, and 1 μs, respectively. The pitch for the plank conduit was 26 nm, and for the bumped conduit 20 nm.

Microwave excitation was sourced from a microwave generator (Anritsu MG3692C) connected via a coaxial cable to a picoprobe (GGB Industries 40A-GSG-150-VP). The picoprobe was positioned using an XYZ micrometer screw stage under an optical microscope to ensure electrical connection the the CPW. The ground-gap-signal widths of the CPWs are 1 μm–1.4 μm–2 μm, respectively, [49].

### 2.2. Characterisation

Atomic force microscopy (AFM) images were taken to characterise the structures’ geometry. Dynamics of spin waves were optically characterised with BLS spectroscopy for (i) thermally excited incoherent spin waves and (ii) electrically excited coherent spin waves. Electrical excitation power was sourced at 5 dBm, compromising between signal strength while still remaining in the linear magnon interaction regime. An area plot was measured with 20 dBm microwave power at source.

**Figure 2:**
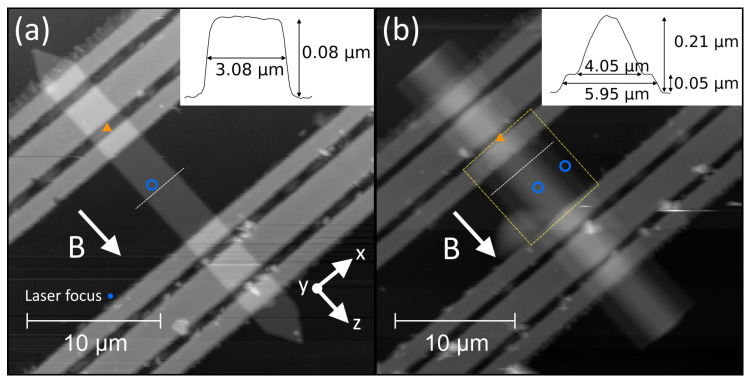
AFM micrographs of both structures. (**a**) 2D sample (plank conduit) of thickness 0.08 μm and width 3.08 μm. (**b**) 3D sample (bumped conduit), the heights of the shoulders and the bump apex are 0.05 μm and 0.21 μm, respectively. Insets show the cross-sectional height profiles at the positions marked by the dotted lines. Circles (triangles) indicate the BLS laser position for each measurement under thermal (microwave) excitation. A scale dot showing the laser focus spot size is indicated on (**a**). An area scan over the boxed region of (**b**) was recorded to investigate the spatial distribution of the propagating spin-wave signal. The external magnetic field is oriented in the substrate plane along the conduits’ long axes, as indicated by the arrows.

The magnonic conduits are composed of approx. 80 at.% Co-Fe embedded in a carbonaceous matrix [50]. The conduits have a prism geometry, i.e., possess translational symmetry along the primary sample axis. The 2D sample is referred to as the plank conduit and the 3D sample is referred to as the bumped conduit. AFM micrographs of both samples are shown in Figure 2.

BLS is the inelastic scattering of light from spin waves in a magnetic material. A schematic representation of our setup is shown in Figure 1b, showing the optical path from the laser source. The laser first passes through some optical components which are present to enhance the single mode nature of the beam and shape the beam for optimal signal strength. The polarised beam splitter above the microscope objective serves to filter out phonon scattered photons, as a distinctive 90° polarisation rotation is acquired upon magnon interaction which is not true for phonon interactions [51]. The microscope objective focuses the laser on the sample, which is situated between the poles of an electromagnet. The back reflected signal is guided to the input of the tandem Fabry–Pérot interferometer (Table Stable TFP-2 HC) to perform high-contrast, high-resolution spectroscopy. The first beam splitter separates a small portion of the beam which is guided to the interferometer and used as a reference. The photons create (Stokes process) or annihilate (anti-Stokes process) magnons, hence the photon frequency decreases or increases, respectively. The Stokes process is more common, and is reported in this work as the absolute frequency shift; a diagram of the interaction is presented in the inset of Figure 1b. Due to energy and momentum conservation, it follows that $\omega_{signal}=\omega_{incident}-\omega_{spinwave}$, hence by measurement of the frequency shift of the inelastically scattered photons with respect to a reference beam component, the magnon mode frequencies are reported. Since the frequency shift of inelastically scattered light is small, a highly monochromatic light source is required such that the frequency-shifted photons are not hidden within a broad spectral peak. Combining a well-tuned laser source with a high-contrast interferometer, a spectroscopic analysis of the inelastically scattered light is performed. In micro-focused BLS, as we have used, a microscope objective focuses the laser, providing sub-micrometer spatial resolution [51]. Our setup uses a 457 nm continuous wave laser, approximately 1 mW of laser power is incident to the sample, trading off between signal strength and the effects of both sample heating and optical pumping of magnetisation dynamics. The beam waist at the focus on the sample position is approximately 400 nm full width at half maximum.

All measurements were taken in the backward volume magnetostatic spin wave geometry, with the external field applied in the plane parallel to the long axis of the conduits, defining the +z direction [5].

Ferromagnetic resonance (FMR) describes the coherent, in-phase, collective, uniform precession of spins at a resonant frequency. The resonant frequency varies with the applied field according to the following relation [52]

(1) $$f_{\mathrm{FMR}}=\frac{\gamma }{2\pi }\sqrt{\left(H-H_{\mathrm{ani}}\right)\left(H-4\pi M_{s}\right)}$$

where $f_{\mathrm{FMR}}$ is the resonant frequency, γ is the gyromagnetic ratio, *H* is the external magnetic field, $H_{\mathrm{ani}}$ is the anisotropy field, and $M_{s}$ is the saturation magnetisation. Because the as-deposited FEBID material is nano-crystalline, crystallographic and strain induced anisotropies are neglected due to the random orientation of the crystallites [50], and shape anisotropy is the dominant anisotropy contribution. In what follows, we present the results of BLS spectroscopic investigation of the FMR modes of the nanostructured waveguides.

## 3. Results

### 3.1. Thermal FMR

**Figure 3:**
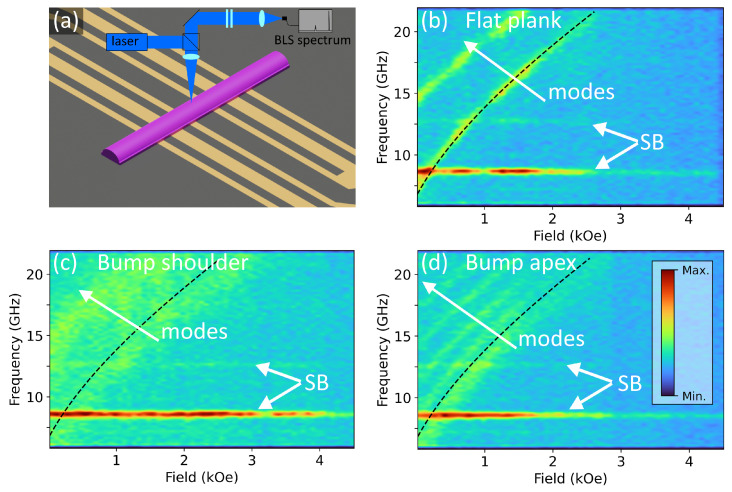
(**a**) Design schematic for the bumped conduit with the *z* dimension stretched for visibility, with simplified BLS operation schematic. Frequency-field plots of the thermally excited BLS-FMR measurements of the flat plank conduit (**b**), the bumped conduit at the shoulder (**c**), and the bump apex (**d**). The constant-frequency intensity peaks at 8.6 GHz and 12.7 GHz are laser side bands, as indicated. Mode number increases with increasing frequency for a given field. The colour scale indicates BLS intensity for all plots, normalised to the highest value in each plot.

The samples’ magnonic thermal spectra were probed by BLS spectroscopy at room temperature. Thermal excitation is spatially uniform and incoherent, thus the BLS signal contains contributions from all magnon modes [53]. The Stokes (magnon creation) BLS spectrum was recorded for external field values from 0 to 4.5 kOe. The BLS spectra for the thermal measurements are reported in Figure 3. Measurement positions are indicated in Figure 2 by blue circles, one position transversely centred on the plank conduit, and one position on each the bump apex and flat shoulder of the bumped conduit.

### 3.2. Microwave Excitation

**Figure 4:**
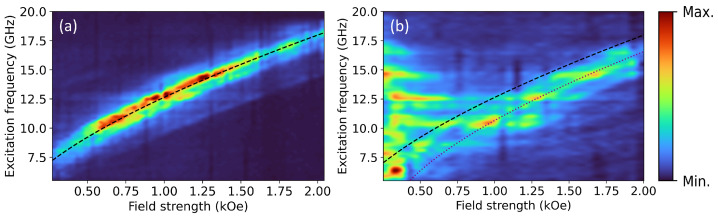
BLS data for a k≠0 mode under local microwave excitation from the CPW for the flat plank conduit (**a**), and the bumped conduit (**b**) measured at the bump apex; positions marked by triangles on Figure 2. A fit of the plank conduit peak positions to Equation (1) has been used to plot the dashed line on both panels. The dotted line on panel (**b**) differs only by the anisotropy field value. The colour scale indicates BLS intensity, normalised to the highest value in each plot.

One frequency-field plot is reported for each sample in Figure 4. These plots show the data for each sample under local excitation by a CPW at one end of the conduit in order to probe the propagating spin–wave dynamics.

Measurement positions are indicated by orange triangles in Figure 2, laterally centred on each conduit and longitudinally placed between the signal and ground lines of the driving CPW. This location was chosen after some preliminary spectra were measured in order to maximise the signal strength. The use of the backward volume geometry means the equilibrium spin orientation will be approximately collinear with the sample long axis (shape and curvature induced anisotropies may result in off-axis magnetisation components in equilibrium). At the measurement position, the CPW Oersted field will be oriented orthogonal to the equilibrium spin orientation, providing the greatest coupling strength, explaining why this is where we observe the greatest coupling strength.

The microwave excitation improves the signal strength, though due to the small size of the antenna and opaque nature of the waveguide material it is not possible to measure a true FMR mode with wavenumber k=0. The external magnetic field was swept from zero to 2 kOe, and microwave power was sourced at 5 dBm.

### 3.3. Area Scan

**Figure 5:**
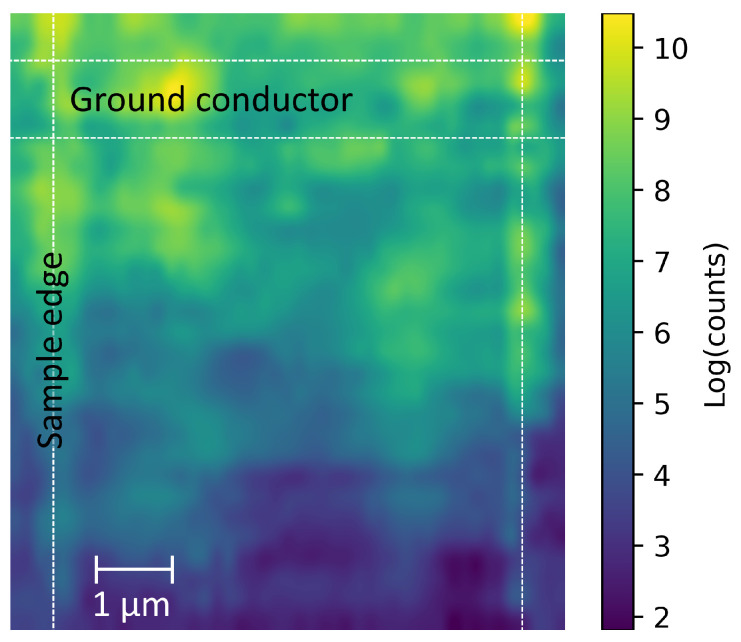
BLS colour map: 2D area plot of the bumped conduit over the region indicated in Figure 2. Signal has been corrected for reflectivity changes due to the surface curvature. Microwave pumping was applied for this measurement though the CPW at the top edge of the figure, with the ground conductor labelled. Colour scale indicates the logarithm of the BLS counts of the integrated signal peak normalised by the reflectivity. Vertical dashed lines show the side edges of the FEBID conduit, the horizontal dashed lines indicate the position of the lower CPW ground conductor.

To understand the spatial intensity distribution of propagating spin waves, a 2D area scan was performed with microwave excitation from one CPW, shown in Figure 5. The bumped conduit was raster scanned under the fixed laser position in order to probe the spin–wave intensity at different positions on the conduit. We expect that the thickness variation of the bumped conduit would result in a non-uniform distribution, due to a modification of the internal field caused by the graduated thickness in the transverse direction [21]. This scan was performed at 13 GHz with a 1.2 kOe external field, chosen to maximise the signal strength. Microwave power was sourced at 20 dBm to increase signal strength.

## 4. Discussion

The lowest order resonance mode for the plank conduit is seen in Figure 3b as the lower curved line of high signal intensity. The line of high intensity signal above this on the plot is due to the first perpendicular standing spin wave (PSSW) mode [54]. The constant-frequency high intensity lines at 8.6 GHz and 12.7 GHz are laser side bands (SB), and unrelated to any magnetic behaviour. Thermal excitation results in a low signal to noise ratio, however as it does not use an antenna, the spectral excitation efficiency is uniform. Because no CPW is used for the thermal measurements, we expect there to be no influence of the antenna on the measurement results. The homogeneous excitation of spin–wave dynamics by thermal pumping [53] results in the measurement primarily probing the dynamics generated directly at the measurement position; dynamics generated away from the measurement position will suffer attenuation before they may be observed. While the CPWs are not excited, they may act as inductively coupled spin wave sinks. However, the measurement position is between the two CPWs, and by considering the propagation length shown in Figure 5 it is clear that the CPWs are sufficiently far from the measurement point that they will have negligible influence on the observed dynamics. The regions of the conduits above the CPWs and the region observed in the thermal measurements are mutually inaccessible for spin waves.

It is expected that the frequency-swept resonance peak in the BLS spectrum for any particular field value has a Lorentzian line shape [55], as was confirmed in the experiment. The low signal amplitude under thermal excitation requires a long acquisition time (ca. 16–20 h). The laser side bands can be seen on Figure 3 to decrease in amplitude at higher field values, also exhibiting instabilities, such as on panel (b). The sideband signal has no dependence on field strength, but field values were varied sequentially starting from zero. The loss of intensity at high field values is, therefore, an indication of the loss of the good alignment condition after long acquisition times. Random drifts in the positioning of optical components over long acquisition times are additive, eventually they surpass the compensatory ability of automatic optimisation routines, due to reaching the travel limit of the piezo positioners. Instabilities are caused by transient effects, such as vibrations.

There are similarities in the thermal frequency-field plots in Figure 3 for both the plank conduit, panel (b), and the shoulder of the bumped conduit, panel (c), in the position of the lowest order resonance mode and the first PSSW mode. The spectrum of the bumped conduit’s shoulder is more broad, however there are distinguishable regions of high intensity which are in similar positions on the frequency-field plot as the well defined modes on the plank conduit plot. This is due to the bumped conduit behaving as a combination of a flat plank and a half cylinder. The modes seen in the shoulder region are similar to those we would observe for a flat plank with the same footprint and thickness equal to the shoulder height. There is some modification due to anisotropy and demagnetising field contributions from the half cylinder.

The bump apex shows many higher order PSSW modes, as shown in Figure 3d. There is an inverse square relation between the PSSW mode spacing and the material thickness [56]; due to the factor of four larger thickness at the bump apex we see that the mode separation significantly decreases, and so more modes exist within the frequency-field space probed (identical for both samples). In further work it will be interesting to analyse the evolution of the mode spectrum with the transverse BLS laser focus position on curved structures and flat structures with graduated thickness (wedges) to determine if this transition in the PSSW mode spectrum is due to only thickness variation, or if this originates from geometric factors, particularly local curvature. We expect that the modification of the PSSW mode spectrum would be continuous for a continuous thickness transition.

Figure 4 showcases our primary finding: the plank conduit supports a single mode under microwave excitation within the frequency-field domain probed, whereas the bumped conduit supports many higher-order modes, demonstrating many modes in the low field regime. The cause of the low field mode abundance is not well understood, but could have a basis in the material not being magnetised to saturation at these lower field values. In this condition, a multi-domain state would exist in which the different domain orientation allows coupling of the CPW Oersted field to higher-order modes, hence allowing their observation. Another possible origin is the variation of the local shape anisotropy. Similar to the above suggested work on the evolution of the PSSW mode spectrum with thickness variation, it would also be enlightening to investigate the evolution of the mode spectrum with a continuous geometric transformation, from a plank-like conduit to a bump-like conduit, with particular attention to the low-field high-frequency domain.

There is an observable horizontal broadening of the low-field higher-frequency modes for the bumped conduit in Figure 4b. The cause of this is not well understood, however, similarly to the instabilities in the laser side bands, it may be related to the stability of the optical system. The distinct differences between Figure 3d and Figure 4b in similar field-frequency regions is striking, and does not well fit the expectation of a simple mode selectivity due to antenna coupling efficiency effects, which we do observe for the flat plank conduit, see Figure 3b and Figure 4a. It is an interesting proposition for further work to test the reproducibility of the horizontal broadening phenomena.

The data for the plank conduit under microwave excitation, shown in Figure 4a, has been fitted using the procedure described above. The frequency swept intensity data were fitted to a Lorentzian line shape, yielding the peak positions and line widths. The higher signal to noise ratio due to the microwave excitation resulted in converging fits for all field positions. The FMR points were fitted to Equation (1) describing the resonance relation, taking γ, $H_{\mathrm{ani}}$, and $M_{s}$ as fitting parameters. γ was bounded within the interval 3.04–3.05 MHz Oe^−1^, in consideration of literature values [57]. The other parameters were freely varying, resulting in γ = 3.04 MHz Oe^−1^, $H_{\mathrm{ani}}$ = −110 Oe, $M_{s}$ = 1159 kA m^−1^. The magnetisation value is close to previously reported values for Co_3_Fe-FEBID nanostructures [21]. With a ca. 80 at.% of Co-Fe in the FEBID deposit, the corrected $M_{s}$ for the Co-Fe deposit amounts to 1449 kA m^−1^. This estimate is in line with the $M_{s}$ value resulting from the expected Co_3_Fe composition with reference values of magnetisation for cobalt of 1400 kA m^−1^ and iron 1700 kA m^−1^, which corresponds to a net magnetisation of 1475 kA m^−1^.

A plot of Equation (1) with the fitted material parameters is shown on both panels of Figure 4 by a black dashed line. A second fit line is shown on panel b with the anisotropy field changed to $H_{\mathrm{ani}}\;=\;$ 210 Oe. The magnetisation and exchange constant are expected to be the same for both structures, due to the near identical deposition parameters. The anisotropy field value was varied as a fitting parameter with accuracy of ±5 Oe.

The Gilbert damping could not be found from the linewidths using the technique described in Reference [55]. This is due to other contributions dominating the intrinsic linewidth. The extracted linewidths for the plank conduit are distributed between approximately 300–600 Oe, which is several times larger than typical linewidths deduced for FEBID nanovolcanos in previous work [42]. The frequency dependence of the linewidth does not exhibit a linear dependence, thus making deduction of the Gilbert damping coefficient impossible.

Despite the lack of a quantitative measure of the spin–wave damping in the direct–write conduits, the 2D area map of the bumped conduit shown in Figure 5 demonstrates the rapid decay of the excited signal as it propagates along the conduit. Some localisation may be seen along the corners of the conduit, which could have a basis in the increased local curvature along each of the edges similar to the results found for some modes in hexagonal nanotubes [22]. The intensity data shown in Figure 5 have been compensated to account for possible effects of the surface curvature of the conduit causing non-uniform reflectivity; a non-magnonic side mode of the laser was used as a normalisation reference. Evident on the area plot is a rapid attenuation of the signal as it propagates away from the exciting antenna indicated at the top edge of the figure. Other work on CoFe FEBID deposits have found a spin–wave decay length of 5–7 μm [21,42], we expect that the rapid attenuation we observed is due to oxidation of the sample. This opens an avenue of investigation into the use of capping materials such as FEBID-NbC which could be placed above the CoFe material as a protective layer to prevent oxidation and improve the lifetime of a CoFe FEBID device.

Finally, we compare the thermal and microwave excitation of the magnonic conduits. The microwave excitation scheme has two primary drawbacks compared to the thermal excitation. (i) The driving Oersted field is oriented in a single direction across the conduit at any time instant, hence only odd modes are excited, as even modes have cancelling contributions across the conduit’s width. For the same reason, excitation efficiency of odd modes decreases with increasing mode number, as a larger proportion of the conduit width has cancelling contributions under a unidirectional driving field. This phenomenon is not present under incoherent thermal excitation, allowing even modes and higher-order odd modes to be detected. (ii) The samples in this work are large compared to the CPW microwave antenna size, as such the antenna’s Oersted field will necessarily be inhomogeneous over the full sample volume. In consequence, under microwave excitation the magnetic moments near the antenna will not precess in phase with those far from the antenna, resulting in a k≠0 mode.

In all, observed differences in the spin–wave transmission should be taken into account during the design of magnonic waveguides. Namely, for multi-mode operation, 3D magnonic conduits support multiple modes which are distinct but closer together in the frequency domain than 2D conduits. In a data carrying application with each mode as a separate channel, this reduces the maximum frequency which must be accessed for a given number of channels, reducing the cost and complexity of associated RF electronics components. For single mode operation, 2D conduits offer a simpler operation regime; as the higher order modes are spaced further from the lowest mode in frequency space compared to 3D conduits, the 2D device is more robust to inaccuracy or imprecision for the driving RF field, allowing less strict engineering tolerances on associated electronic devices. The greater spacing also reduces the energy lost to inter-mode scattering. While there is no available analytical theory at hand for the quantitative description of the magnon dispersion in 3D magnetic nano-architectures, our findings show a clear difference in the spin-wave spectra for 2D and 3D magnonic conduits. We anticipate that the direct writing of magnonic conduits by FEBID can be extended toward structures with thickness variation.

## 5. Conclusions

In summary, we have demonstrated the ability to fabricate magnetic 3D Co_3_Fe nanostructures by direct write using FEBID which show a significant change in the supported magnon spectra. The richness of the magnon spectra is attributed to field non-uniformity induced effects which are derived solely from the 3D geometry of the structures. Our observations stimulate further investigations into the use of geometric engineering in magnetic structures to deliberately tune the supported mode spectra of magnonic conduits. Further investigations of 3D geometry and curvature effects in conduit structures from a theoretical or numeric approach would guide the direction of further experimental studies of complex-shaped magnonic conduits.

## Data Availability

The data presented in this study are available on reasonable request from the corresponding author.

## References

[1] Barman A., Gubbiotti G., Ladak S., Adeyeye A.O., Krawczyk M., Grafe J., Adelmann C., Cotofana S., Naeemi A., Vasyuchka V.I. (2021). The 2021 Magnonics Roadmap. J. Physics Condens. Matter.

[2] Chumak A.V., Kabos P., Wu M., Abert C., Adelmann C., Adeyeye A.O., Akerman J., Aliev F.G., Anane A., Awad A. (2022). Advances in Magnetics Roadmap on Spin-Wave Computing. IEEE Trans. Magn..

[3] Wang Q., Kewenig M., Schneider M., Verba R., Kohl F., Heinz B., Geilen M., Mohseni M., Lägel B., Ciubotaru F. (2020). A magnonic directional coupler for integrated magnonic half-adders. Nat. Electron..

[4] Zakeri K. (2018). Terahertz magnonics: Feasibility of using terahertz magnons for information processing. Phys. C Supercond. Appl..

[5] Chumak A.V., Serga A.A., Hillebrands B. (2014). Magnon transistor for all-magnon data processing. Nat. Commun..

[6] Dobrovolskiy O.V., Sachser R., Bunyaev S.A., Navas D., Bevz V.M., Zelent M., Śmigaj W., Rychły J., Krawczyk M., Vovk R.V. (2019). Spin-Wave Phase Inverter upon a Single Nanodefect. ACS Appl. Mater. Interfaces.

[7] Fischer T., Kewenig M., Bozhko D.A., Serga A.A., Syvorotka I.I., Ciubotaru F., Adelmann C., Hillebrands B., Chumak A.V. (2017). Experimental prototype of a spin-wave majority gate. Appl. Phys. Lett..

[8] Qin H., Holländer R.B., Flajšman L., Hermann F., Dreyer R., Woltersdorf G., van Dijken S. (2021). Nanoscale magnonic Fabry-Pérot resonator for low-loss spin-wave manipulation. Nat. Commun..

[9] Yuan H.Y., Cao Y., Kamra A., Duine R.A., Yan P. (2022). Quantum magnonics: When magnon spintronics meets quantum information science. Phys. Rep..

[10] Mohseni M., Vasyuchka V.I., L’vov V.S., Serga A.A., Hillebrands B. (2022). Classical analog of qubit logic based on a magnon Bose–Einstein condensate. Commun. Phys..

[11] Andrianov S.N., Moiseev S.A. (2014). Magnon qubit and quantum computing on magnon Bose–Einstein condensates. Phys. Rev. A At. Mol. Opt. Phys..

[12] Grollier J., Querlioz D., Camsari K.Y., Everschor-Sitte K., Fukami S., Stiles M.D. (2020). Neuromorphic spintronics. Nat. Electron..

[13] Demokritov S.O., Demidov V.E., Dzyapko O., Melkov G.A., Serga A.A., Hillebrands B., Slavin A.N. (2006). Bose–Einstein condensation of quasi-equilibrium magnons at room temperature under pumping. Nature.

[14] Schneider M., Breitbach D., Serha R.O., Wang Q., Serga A.A., Slavin A.N., Tiberkevich V.S., Heinz B., Lägel B., Brächer T. (2021). Control of the Bose–Einstein Condensation of Magnons by the Spin Hall Effect. Phys. Rev. Lett..

[15] Wang Q., Chumak A.V., Pirro P. (2021). Inverse-design magnonic devices. Nat. Commun..

[16] Papp A., Porod W., Csaba G. (2021). Nanoscale neural network using non-linear spin-wave interference. Nat. Commun..

[17] Vogel M., Chumak A.V., Waller E.H., Langner T., Vasyuchka V.I., Hillebrands B., Von Freymann G. (2015). Optically reconfigurable magnetic materials. Nat. Phys..

[18] Dobrovolskiy O.V., Sachser R., Brächer T., Böttcher T., Kruglyak V.V., Vovk R.V., Shklovskij V.A., Huth M., Hillebrands B., Chumak A.V. (2019). Magnon–fluxon interaction in a ferromagnet/superconductor heterostructure. Nat. Phys..

[19] Xu M., Yamamoto K., Puebla J., Baumgaertl K., Rana B., Miura K., Takahashi H., Grundler D., Maekawa S., Otani Y. (2020). Nonreciprocal surface acoustic wave propagation via magneto-rotation coupling. Sci. Adv..

[20] Papp A., Kiechle M., Mendisch S., Ahrens V., Sahin L., Seitner L., Porod W., Csaba G., Becherer M. (2021). Experimental demonstration of a concave grating for spin waves in the Rowland arrangement. Sci. Rep..

[21] Dobrovolskiy O.V., Vovk N.R., Bondarenko A.V., Bunyaev S.A., Lamb-Camarena S., Zenbaa N., Sachser R., Barth S., Guslienko K.Y., Chumak A.V. (2021). Spin-wave eigenmodes in direct-write 3D nanovolcanoes. Appl. Phys. Lett..

[22] Körber L., Zimmermann M., Wintz S., Finizio S., Kronseder M., Bougeard D., Dirnberger F., Weigand M., Raabe J., Otálora J.A. (2021). Symmetry and curvature effects on spin waves in vortex-state hexagonal nanotubes. Phys. Rev. B.

[23] Li X., Labanowski D., Salahuddin S., Lynch C.S. (2017). Spin wave generation by surface acoustic waves. J. Appl. Phys..

[24] Heinz B., Brächer T., Schneider M., Wang Q., Lägel B., Friedel A.M., Breitbach D., Steinert S., Meyer T., Kewenig M. (2020). Propagation of Spin-Wave Packets in Individual Nanosized Yttrium Iron Garnet Magnonic Conduits. Nano Lett..

[25] Wang Q., Heinz B., Verba R., Kewenig M., Pirro P., Schneider M., Meyer T., Lägel B., Dubs C., Brächer T. (2019). Spin Pinning and Spin-Wave Dispersion in Nanoscopic Ferromagnetic Waveguides. Phys. Rev. Lett..

[26] Gubbiotti G. (2019). Three-Dimensional Magnonics.

[27] Garlando U., Wang Q., Dobrovolskiy O.V., Chumak A.V., Riente F. (2023). Numerical Model for 32-bit Magnonic Ripple Carry Adder. arXiv.

[28] May A., Saccone M., van den Berg A., Askey J., Hunt M., Ladak S. (2021). Magnetic charge propagation upon a 3D artificial spin-ice. Nat. Commun..

[29] Ho P., Tan A.K., Goolaup S., Oyarce A.L., Raju M., Huang L.S., Soumyanarayanan A., Panagopoulos C. (2019). Geometrically tailored skyrmions at zero magnetic field in multilayered nanostructures. Phys. Rev. Appl..

[30] Sheka D.D. (2021). A perspective on curvilinear magnetism. Appl. Phys. Lett..

[31] Makarov D., Volkov O.M., Kákay A., Pylypovskyi O.V., Budinská B., Dobrovolskiy O.V., Makarov D., Volkov O.M., Kákay A., Pylypovskyi O.V. (2022). New Dimension in Magnetism and Superconductivity: 3D and Curvilinear Nanoarchitectures. Adv. Mater..

[32] Fernández-Pacheco A., Streubel R., Fruchart O., Hertel R., Fischer P., Cowburn R.P. (2017). Three-dimensional nanomagnetism. Nat. Commun..

[33] Smith E.J., Makarov D., Sanchez S., Fomin V.M., Schmidt O.G. (2011). Magnetic microhelix coil structures. Phys. Rev. Lett..

[34] Sanz-Hernández D., Hamans R.F., Osterrieth J., Liao J.W., Skoric L., Fowlkes J.D., Rack P.D., Lippert A., Lee S.F., Lavrijsen R. (2018). Fabrication of Scaffold-Based 3D Magnetic Nanowires for Domain Wall Applications. Nanomaterials.

[35] Harinarayana V., Shin Y.C. (2021). Two-photon lithography for three-dimensional fabrication in micro/nanoscale regime: A comprehensive review. Opt. Laser Technol..

[36] Fernández-Pacheco A., Skoric L., De Teresa J.M., Pablo-Navarro J., Huth M., Dobrovolskiy O.V. (2020). Writing 3D Nanomagnets Using Focused Electron Beams. Materials.

[37] Höflich K., Hobler G., Allen F.I., Wirtz T., Rius G., Krasheninnikov A.V., Schmidt M., Utke I., Klingner N., Osenberg M. (2023). Roadmap for focused ion beam technologies. arXiv.

[38] Weitzer A., Huth M., Kothleitner G., Plank H. (2022). Expanding FEBID-Based 3D-Nanoprinting toward Closed High-Fidelity Nanoarchitectures. ACS Appl. Electron. Mater..

[39] Córdoba R., Orús P., Strohauer S., Torres T.E., De Teresa J.M. (2019). Ultra-fast direct growth of metallic micro- and nano-structures by focused ion beam irradiation. Sci. Rep. X.

[40] Dobrovolskiy O.V., Begun E., Bevz V.M., Sachser R., Huth M. (2020). Upper Frequency Limits for Vortex Guiding and Ratchet Effects. Phys. Rev. Appl..

[41] Heil T., Waldow M., Capelli R., Schneider H., Ahmels L., Tu F., Schoneberg J., Marbach H. (2021). Pushing the limits of EUV mask repair: Addressing sub-10 nm defects with the next generation e-beam-based mask repair tool. J. Micro/Nanopatterning Mater. Metrol..

[42] Bunyaev S.A., Budinska B., Sachser R., Wang Q., Levchenko K., Knauer S., Bondarenko A.V., Urbánek M., Guslienko K.Y., Chumak A.V. (2021). Engineered magnetization and exchange stiffness in direct-write Co–Fe nanoelements. Appl. Phys. Lett..

[43] Urbánek M., Flajšman L., Křiáková V., Gloss J., Horký M., Schmid M., Varga P. (2018). Research Update: Focused ion beam direct writing of magnetic patterns with controlled structural and magnetic properties. APL Mater..

[44] Davies C.S., Francis A., Sadovnikov A.V., Chertopalov S.V., Bryan M.T., Grishin S.V., Allwood D.A., Sharaevskii Y.P., Nikitov S.A., Kruglyak V.V. (2015). Towards graded-index magnonics: Steering spin waves in magnonic networks. Phys. Rev. B Condens. Matter Mater. Phys..

[45] Gruszecki P., Krawczyk M. (2018). Spin-wave beam propagation in ferromagnetic thin films with graded refractive index: Mirage effect and prospective applications. Phys. Rev. B.

[46] Kiechle M., Papp A., Mendisch S., Ahrens V., Golibrzuch M., Bernstein G.H., Porod W., Csaba G., Becherer M., Kiechle M. (2023). Spin-Wave Optics in YIG Realized by Ion-Beam Irradiation. Small.

[47] Lara A., Dobrovolskiy O.V., Prieto J.L., Huth M., Aliev F.G. (2014). Magnetization reversal assisted by half antivortex states in nanostructured circular cobalt disks. Appl. Phys. Lett..

[48] Khitun A., Bao M., Wang K.L. (2010). Magnonic logic circuits. J. Phys. D Appl. Phys..

[49] Vaňatka M., Szulc K., Wojewoda O., Dubs C., Chumak A.V., Krawczyk M., Dobrovolskiy O.V., Kłos J.W., Urbánek M. (2021). Spin-Wave Dispersion Measurement by Variable-Gap Propagating Spin-Wave Spectroscopy. Phys. Rev. Appl..

[50] Porrati F., Pohlit M., Müller J., Barth S., Biegger F., Gspan C., Plank H., Huth M. (2015). Direct writing of CoFe alloy nanostructures by focused electron beam induced deposition from a heteronuclear precursor. Nanotechnology.

[51] Sebastian T., Schultheiss K., Obry B., Hillebrands B., Schultheiss H. (2015). Micro-focused Brillouin light scattering: Imaging spin waves at the nanoscale. Front. Phys..

[52] Kakazel G.N., Wigen P.E., Guslienko K.Y., Chantrell R.W., Lesnik N.A., Metlushko V., Shima H., Fukamichi K., Otani Y., Novosad V. (2003). In-plane and out-of-plane uniaxial anisotropies in rectangular arrays of circular dots studied by ferromagnetic resonance. J. Appl. Phys..

[53] Lendinez S., Taghipour Kaffash M., Jungfleisch M.B. (2021). Observation of mode splitting in artificial spin ice: A comparative ferromagnetic resonance and Brillouin light scattering study. Appl. Phys. Lett..

[54] Kittel C. (1958). Excitation of Spin Waves in a Ferromagnet by a Uniform rf Field. Phys. Rev..

[55] Kalarickal S.S., Krivosik P., Wu M., Patton C.E., Schneider M.L., Kabos P., Silva T.J., Nibarger J.P. (2006). Ferromagnetic resonance linewidth in metallic thin films: Comparison of measurement methods. J. Appl. Phys..

[56] Schreiber F., Frait Z. (1996). Spin-wave resonance in high-conductivity films: The Fe-Co alloy system. Phys. Rev. B.

[57] Schoen M.A., Lucassen J., Nembach H.T., Silva T.J., Koopmans B., Back C.H., Shaw J.M. (2017). Magnetic properties of ultrathin 3d transition-metal binary alloys. I. Spin and orbital moments, anisotropy, and confirmation of Slater-Pauling behavior. Phys. Rev. B.

